# Physicians' self-assessed empathy levels do not correlate with patients' assessments

**DOI:** 10.1371/journal.pone.0198488

**Published:** 2018-05-31

**Authors:** Monica Oliveira Bernardo, Dario Cecílio-Fernandes, Patrício Costa, Thelma A. Quince, Manuel João Costa, Marco Antonio Carvalho-Filho

**Affiliations:** 1 Radiology Department–Faculty of Medicine—Catholic University of São Paulo–Sorocaba–São Paulo—Brazil; 2 Center for Educational Development Innovation and Research–University Medical Center–University of Groningen–Groningen–The Netherlands; 3 Life and Health Sciences Research Institute—School of Health Sciences—University of Minho–Braga–Portugal; 4 Department of Public Health and Primary Care–University of Cambridge–Cambridge—United Kingdom; 5 Life and Health Sciences Research Institute—School of Health Sciences—University of Minho–Braga–Portugal; 6 Emergency Department–School of Medical Sciences–University of Campinas–Campinas–São Paulo–Brazil; 7 Center for Educational Development Innovation and Research–University Medical Center–University of Groningen–Groningen–The Netherlands; Florida International University, UNITED STATES

## Abstract

**Background:**

Empathy is a fundamental humanistic component of patient care which facilitates efficient and patient-centered clinical encounters. Despite being the principal recipient of physician empathy little work on how patients perceive/report receiving empathy from their physicians has been undertaken. In the context of doctor-patient interactions, knowledge about empathy has mostly originated from physicians’ perspectives and has been developed from studies using self-assessment instruments. In general, self-assessment may not correlate well with the reality observed by others.

**Objectives:**

To investigate: 1—the relationship between physicians’ self-assessed empathy and patients’ measures of physicians’ empathy; 2 –Environmental factors that could influence patients’ perceptions; and 3 –the correlation between two widely used psychometric scales to measure empathy from the perspective of patients.

**Methods:**

This is an observational study which enrolled 945 patients and 51 physicians from radiology, clinical, and surgical specialties. The physicians completed the Jefferson Scale of Physician Empathy (JSE) and the International Reactivity Index (IRI), and patients completed the Consultation and Relational Empathy scale (CARE), and the Jefferson Scale of Patient’s Perceptions of Physician Empathy (JSPPPE).

**Results:**

We did not observe any significant correlation between total self-assessed empathy and patients’ perceptions. We observed a small correlation (r = 0,3, P<0,05) between the sub-dimension Perspective Taking-JSE and JSPPPE. JSPPPE and CARE had a positive and moderate correlation (0,56; p<0,001). Physicians’ gender and sector influenced the JSPPPE score. Sector, medical specialty and the nature of the appointment (initial versus subsequent) influenced the CARE measure.

**Conclusions:**

The lack of correlation between self-assessed empathy levels and patients’ perceptions suggests patients be included in the process of empathy evaluation.

**Practice implications:**

Training strategies aiming the development of empathy should include patients’ evaluations and perspectives.

## Introduction

Empathy is a fundamental humanistic component of patient care [1] which facilitates efficient and patient-centered clinical encounters [2,3]. Positive associations have been found between physicians’ self-measured empathy and patients’ outcomes, for example in LDL control and the incidence of metabolic complications of diabetes [4,5]. Moreover, empathetic doctors are more satisfied with their jobs and less susceptible to burnout and depression [6–9]. However, a number of problems surround the definition, components, and measurement of empathy [10].

Empathy is multidimensional, involving affective, cognitive and behavioral components [11]. The affective component refers to one’s ability to perceive subjectively another person’s inner experiences and natural feelings [12]. The cognitive component of empathy relates to the capacity to understand and view the outside world from the other person’s perspective [12]. The behavioral component includes the predisposition and competency to adequately create a bond with the other person together with the ability to communicate these understandings and feelings to reassure and comfort the other [13,14].

Despite being the principal recipient of physician empathy little work on how patients perceive/report receiving empathy from their physicians has been undertaken [15]. Indeed, in the context of doctor-patient interactions, knowledge about empathy has mostly originated from physicians’ perspectives and has been developed from studies using self-assessment instruments [16,17]. Very few studies have compared standardized patients’ measures of physicians’ empathy with physicians’ or medical students’ self-assessed empathy. Those undertaken have reported no significant correlations [18,19]. In general, self-assessment may not correlate well with the reality observed by others [20,21]. Measuring physician empathy is also fraught by the variety of instruments employed which research suggests may measure differing constructs [22,23].

Therefore, physicians' view of their own empathy may be at worst incorrect and at best biased. For instance, social expectations about what is considered a desirable attitude for doctors may influence the way physicians appreciate themselves [22]. It would seem crucial, therefore, that any evaluation of physician empathy should consider patients’ perspectives. This would allow a more concrete understanding of physician/patient interaction [23].

However, empowering patients to measure physicians’ empathy is not simple nor easy. There are constraints related to time, and logistics to fit patients’ assessment on routine clinical activities [24]. Moreover, a cultural change is needed to make doctors aware of the need to consolidate the role of the patient as a legitimate evaluator of physicians’ behaviors and attitudes [25].

In addition, patients’ perspectives can also be influenced by different factors beyond the direct interaction with their doctors. These factors include the prevailing physical ambiance, the patient’s or physician’s gender, and the duration or context of the consultation [26,27]. A recent Argentinean study showed that age, education level, South American ascendancy and type of hospital influenced patients’ perceptions of physicians’ empathy [28].

Despite the importance of their views, to the authors’ knowledge, only three studies directly compared physicians’ perceptions of their own empathy and patients’ perceptions of physicians’ empathy, using validated psychometric scales. [29–31]. Two studies reported no significant correlation, but both studies had small samples of physicians (n = 27 and n = 29), and the number of patients was not reported [29,30]. A third study found a positive and significant correlation, but the samples of physicians and patients were small (36 physicians and 90 patients) [31]. Since medical training at both graduate and post-graduate levels relies heavily on self-assessed measures of physicians’ empathy, more studies are needed to understand in what extent self-assessed empathy matches with patients’ perspectives. This understanding would be particularly relevant to guide feedback and further professional development of doctors.

Our study set out to investigate the relationship between physicians’ self-assessed empathy levels and patients’ measures of physicians’ empathy. We also investigated whether patients’ assessments could be influenced by patient socio-demographics and consultation contextual factors in the physician-patient interaction. These factors included gender, medical specialty, and aspects of the consultation, such as location and whether this was an initial or subsequent consultation. This work was undertaken in public and private hospital settings in Brazil.

Based on the assumption that self-assessment is frequently not accurate [20], we hypothesized that self-assessed empathy levels would be poorly correlated with patients’ perceptions. Empathy has several dimensions which may not be fully covered by one instrument. Therefore, we used two instruments that capture different constructs of empathy [22] to measure self-assessed physician empathy and two instruments to measure patients’ perspectives of physicians’ empathy.

For physicians’ self-assessment we used the Jefferson Scale of Empathy (JSE) and the Interpersonal Reactivity Index (IRI). These instruments were developed with different perspectives [32]. The JSE was specifically designed to measure empathy in the healthcare context and focuses on the cognitive aspects of physician empathy [29]. IRI was developed to measure empathy in the general population and assesses both the affective and cognitive components of empathy [27]. An international study demonstrated that these two instruments capture different constructs of empathy [22].

The instruments to evaluate patients’ perspectives of physician empathy were the Jefferson Scale of Patient’s Perceptions of Physician Empathy (JSPPPE) and the Consultation and Relational Empathy Scale (CARE). The JSPPPE was developed assuming empathy to be a “predominantly cognitive attribute” [33].

The CARE measure was developed to capture patients’ expectations of clinical encounters [34,35]. Both instruments were based on extensive literature reviews, and the latter was also based on in-depth interviews with a group of ambulatory patients. We hypothesized that JSPPPE and CARE could also reflect patients’ views of the different components of empathy.

To explore the relationship between patients’ and physicians’ assessments of physician empathy we asked the following research questions:

1. How do scores on patient scales (JSPPPE and CARE) relate to each other?
2. Do the patients’ scores vary according to contextual aspects of the consultation (gender, location, specialty or initial versus subsequent consultations) beyond the specific doctor/patient interaction?
3. Do self-assessed physicians’ scores on the IRI and the JSPE scales correlate with patients’ scores obtained with the JSPPPE and CARE scales?

## Methods

### Participants

#### Physicians

All physicians in two clinics (one private, one public) located in the same multi-specialty medical center in Sorocaba, São Paulo, Brazil (n = 60) were invited to participate, and 51 agreed to do so. Those who declined to participate mentioned time constraints (n = 6) and not feeling comfortable taking part (n = 3). All participants were experienced physicians with an average of 18,8 years (±11,7/range 3–43) years of practice and comprised internists (n = 24), surgeons (n = 10) and radiologists (n = 17). Table 1 present physician’s demographic information, local of work, their specialties, and self-assessed empathy levels (Table 1).

**Table 1:** Descriptive and comparative statistics for physicians’ characteristics.

Physicians’ characteristics		N (%)	JSE	IRI
Gender	Male	35 (69%)	116,3 ± 16,5	57 ± 10,1
	Female	16 (31%)	123,3 ± 9,0	61,6 ± 9,1
Sector	Private	39 (76%)	119,2 ± 12,7	58,3 ± 9,6
	Public	12 (24%)	116,2 ± 20,9	58,9 ± 11,5
Specialty	Internal Medicine	24 (47%)	120,4 ± 11,8	58 ± 10,9
	Surgery	10 (20%)	117,4 ± 23,2	57,3 ± 10,2
	Radiology	17 (33%)	116,4 ± 13,3	59,7 ± 8,8
	Total	51	118,5 ± 14,9	58,4 ± 9,9

JSE = Jefferson Scale of Physician Empathy; IRI = Interpersonal Relative Index.

#### Patients

**Fig 1:**
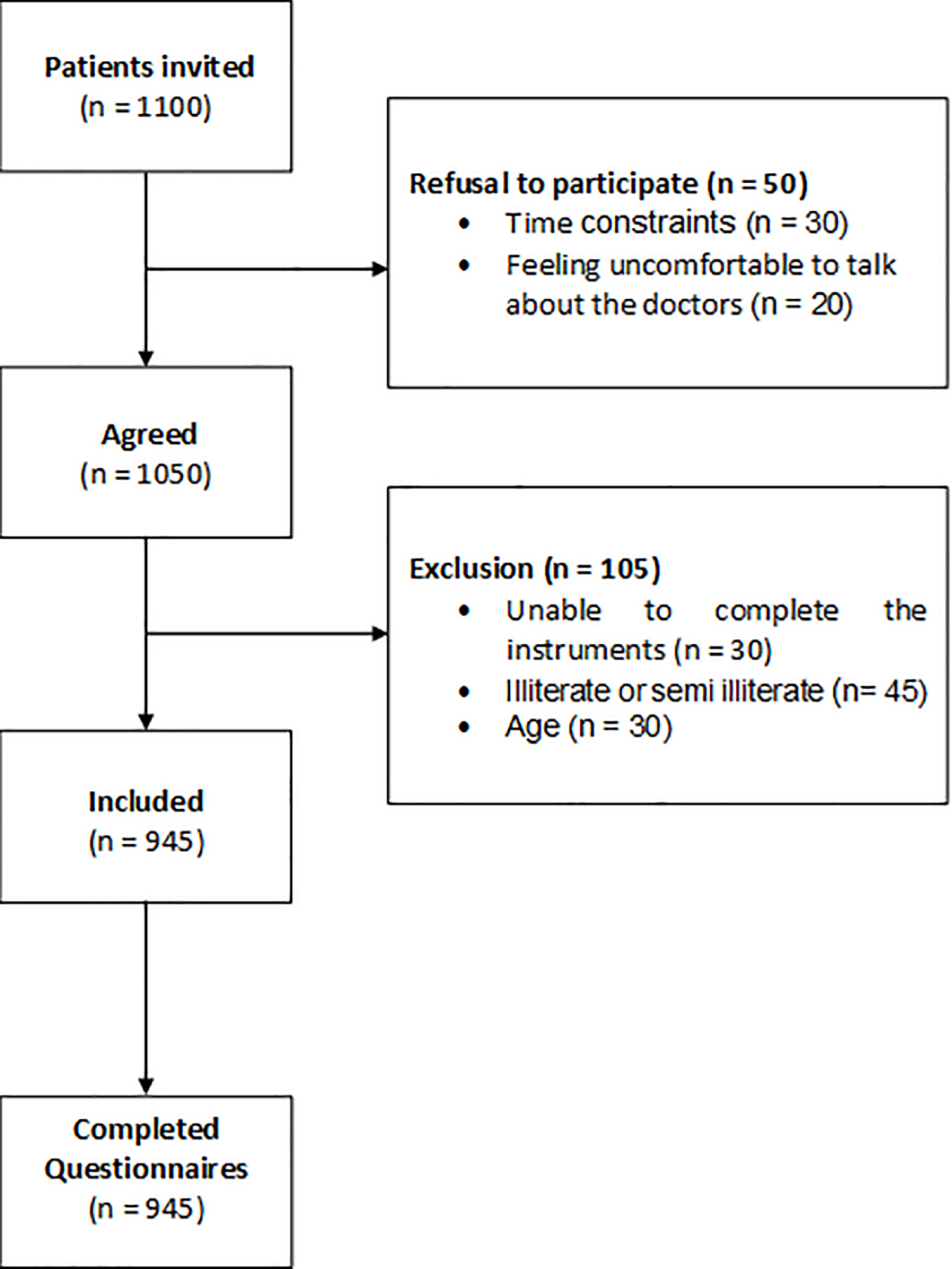
Patient´s flowchart.

Adult patients (n = 1100) under the supervision of the participating physicians were randomly invited to join the study and 1050 decided to participate. Fig 1 gives the patient flow chart: 50 patients declined because time constraints and feeling uncomfortable commenting on their doctors. A further 105 were excluded because they were under 18 years-old, not able to complete the instrument, or illiterate. The total number of patients was 945, of whom 639 were females (average age 50,5 years-old ± 14,1/range 19–84) and 306 males (average age 50,4 years-old ± 14,8/range 18–94). Between 18 and 25 patients of each of the participating doctors took part in the study. (The primary objective was to have 25 patients per doctor.)

### Administration procedures

Paper questionnaires were used and patients were approached by an independent researcher, with no responsibility or connection to their care. The patients were instructed to fill the scales and to hand them back to the researcher in a closed envelope. All forms were anonymized. Patients and physicians had access only to their own aggregate results at the end of the study. The questionnaires were returned directly to the researchers in closed envelopes and the results were inserted into a data system by a designated person, who did not have access to patients’ names.

### Instruments

#### Physicians

We used the Portuguese versions of the Jefferson Scale of Empathy (JSE) and the Interpersonal Reactivity Index (IRI assess physicians’ self-assessment of their empathy. Both self-assessment instruments have a mixture of positive and negative items and both ask respondents to rate the extent to which they agree or disagree with statements. JSE has 20 items each rated on a 7-point Likert scale and comprises three subdimensions, Perspective Taking, Compassionate Care and Standing on the Patients’ shoes [36]. The IRI has 28 items each rated on a 5-point Likert scale and comprises four distinct dimensions: Perspective Taking, Empathic Concern, Personal distress and Fantasy [37].

#### Patients

We used the Jefferson Scale of Patient’s Perceptions of Physician Empathy (JSPPPE) and the Consultation and Relational Empathy scale (CARE) to measure physicians’ empathy as perceived by their patients. Both instruments ask patients to rate the extent they agree or with statements and both are administered at the end of the clinical consultation. The JSPPPE has 5-items rated on a 7-point Likert scale [29,30]. As no Portuguese version was available it was translated into Portuguese by four bilingual physicians and checked for accuracy by a professional translator. The developers of the original English version supervised the process [38,39]. Researchers interviewed voluntary patients (n = 100) to verify whether the final version was understandable. The CARE instrument has 10 items which address different components of empathy (affective, cognitive and behavioral). Each item is measured on a 6-point Likert scale. We used the Portuguese version of CARE scale, which was translated by Scarpellini in 2014 [40]. We performed an analysis of the structure of the scale, as this has not done before.

#### Empathy scores

The total score of JSPPPE and CARE is the sum of all item scores and all the items must be answered to be included. The overall score for the JSE is the sum of all items scores, but the negative items are transformed into positive ones for analyses; the higher the total score, the higher the levels of empathy. Similarly, negative items on the IRI were transformed into positive scores. We used the subscale scores for both the JSE and IRI. In accordance with other studies of physician empathy we used the total score for the JSE. For the purposes of consistency in the analysis we also used the total score for the IRI.

### Statistical analyses

We investigated the psychometric properties of the JSPPPE and CARE instruments. The absolute values of skewness and kurtosis for all items were within the acceptable range of the normal distribution (between -2 and 2) for both JSPPPE and CARE scales, with the exception of item number 5 of the JSPPPE scale. Subsequent analyses demonstrated that there was no difference when using the raw and transformed data in the outcome of the analysis. Therefore, we only present the outcomes of parametric analyses using the raw data. Cross-validation of the JSPPPE and CARE was assessed using a holdout method with Principal Component Analysis and Confirmatory Factor Analysis, applied to two sub-samples (A and B), obtained from randomization of the full sample. An exploratory principal component analysis with Principal Axis Factoring was applied to sub-sample A (n = 474). A confirmatory factor analysis with Maximum Likelihood estimation was applied to sub-sample B (n = 471). To assess the best confirmatory factor model, we used the following goodness of fit: Chi-square statistics, Comparative Fit Index (CFI), Tucker-Lewis Index (TLI) and Root Mean Square Error of Approximation (RMSEA). The Chi-square statistics was used to assess the overall fit and discrepancy between the sample and the model. Both CFI and TLI were considered optimal with values above 0,90. Optimal RMSEA is close to zero. Reliability was calculated using Cronbach Alpha.

We used Pearson correlation to investigate the relation between physicians’ self-report empathy and the empathy perceived by their patients. Since the Jefferson Scale of Empathy (JSE) and the Jefferson Scale of Patient’s Perceptions of Physician Empathy (JSPPPE) were developed by the same researchers using similar words in many questions, we also have analyzed the intra-class correlation between the items of the JSPPPE and JSP. We have analyzed the four from the JSPPPE with items 2 and 10 from JSE; the item 3 from the JSPPPE with item 16 from JSE; and the item 1 from JSPPPE with items 3, 6 and 9 from JSE. We compared patients’ assessments in respect of their gender and sector (public vs. private) using t-tests and we investigated differences between medical specialty by means of an analysis of variance (ANOVA).

Data were analyzed using IBM-SPSS 21.0 and AMOS 18. The latter was only used for the confirmatory factor analysis.

### Ethical approval

We obtained ethical approval for this study from the Research Ethics Committee of the Faculty of Medical Sciences–São Paulo Catholic University in June 2015 (CAAE = 46056115.0.0000.5373). All participants gave written informed consent before data collection began.

## Results

### Cross-validation of instruments

There are no validation studies in the respective contexts of the instruments used in this study to assess physician empathy as perceived by the patient participants. Therefore, we included a validation step.

### JSPPPE

The necessary assumption of Principal Component Analysis (PCA) was met with a KMO = 0,781, and Bartlett’s Test of Sphericity was significant (p <0,001). The PCA demonstrated a unidimensional factorial structure with an eigenvalue of 3,42, explaining 68,9% of the variance; factor coefficients ranged from 0,84 to 0,87.

Confirmatory Factor Analysis (CFA) revealed that the base model (model A) for the **JSPPPE** demonstrated poor fit index values, based on the χ2/df ratio, the Comparative Fit Index (CFI) and Root Mean Square Error of Approximation (RMSEA). When the correlation between the items’ errors was added (model B), the model achieved a satisfactory level of model fit (Table 2).

**Table 2:** Fit indices for the JSPPE.

	χ^2^(df) Sig.	Ratio χ^2^/df	TLI	CFI	RMSEA (HI90)
Model A	χ^2^(5) = 61,38; p<0,001	12,276	0,932	0,966	0,155 (0,191)
Model B	χ^2^(3) = 5,66; p = 0,129	1,888	0,995	0,998	0,043 (0,098)

Cronbach's Alpha for the total sample was 0,88, indicating that the instrument is reliable.

### CARE

The necessary assumptions of PCA were meet with a KMO = 0,849, and Bartlett’s Test of Sphericity was significant (p <0,001). The Principal Component Analysis demonstrated a unidimensional factorial structure with an eigenvalue of 7,66, explaining 76,7% of the variance; factor coefficients ranged from 0,77 to 0,91.

Confirmatory Factor Analysis (CFA) revealed that the base model for the CARE scale (model A) displayed poor fit index values, based on the χ2/df ratio, the Comparative Fit Index (CFI) and Root Mean Square Error of Approximation (RMSEA). When the correlation between the items’ errors was added (model B), the model achieved a satisfactory level of model fit (Table 3).

**Table 3:** Fit indices for the CARE scale.

	χ^2^(df) Sig.	Ratio χ^2^/df	TLI	CFI	RMSEA (HI90)
Model A	χ^2^(35) = 307; p<0,001	8,771	0,940	0,954	0,129 (0,142)
Model B	χ^2^(30) = 126; p<0,001	4,200	0,975	0,984	0,083 (0,098)

Cronbach's Alpha for the total sample was 0,97, indicating that the instrument is reliable.

### Concurrent validity

The correlation between the **JSPPPE** and **CARE** latent variables was moderate (0,56) and significant (p<0,001), indicating that both scales share 32% of the same measurement.

### Contextual elements influencing patients’ perspectives (Table 4)

Patients’ gender did not affect their assessments on either the CARE or the JSPPPE scales. However, patients’ assessments of female physicians were higher on the JSPPPE scale but not on the CARE scale.

**Table 4:** Descriptive and comparative statistics for patient’s characteristics and measurements.

		N	%	JSPPPE ^a^	P	CARE ^b^	P
**Patient’s Gender**	**. Male**	306	32%	30,1 ± 5,8	p = 0,064	43,1 ± 7,5	p = 0,302
	**. Female**	639	68%	30,8 ± 5,4		42,6 ± 7,8	
**Physician's Gender**	**. Male**	692	73%	**30,2 ± 5,7**	**p<0,001**	42,5 ± 7,7	p = 0,124
	**. Female**	253	27%	**31,6 ± 5,2**		43,4 ± 7,8	
**Sector**	**. Private**	810	86%	**31,0 ± 5,1**	**p<0,001**	**43,5 ± 7,3**	**p<0,001**
	**. Public**	135	14%	**28,2 ± 7,4**		**38,2 ± 8,5**	
**Specialty**	**. Internal Medicine**	437		31,1 ± 5,3	p = 0,055	**43,8 ± 7,5**	**p<0,001**
	**. Surgery**	177		30,0 ± 6,0		**42,3 ± 7,8**	
	**. Radiology**	331		30,1 ± 5,7		**41,5 ± 7,8**	
**Appointment**	**. Initial**	543	57%	30,4 ± 5,5	p = 0,266	**42,3 ± 7,6**	**p = 0,031**
	**. Subsequent**	402	43%	30,8 ± 5,7		**43,4 ± 7,8**	
	Total	945	100%	30,6 ± 5,6		42,8 ± 7,7	

JSSPPE = Jefferson Scale of Patients Perceptions of Physician Empathy; CARE = Consultation and Relational Empathy Scale.

Patients in the private sector perceived their physicians’ empathy to be significantly higher than those in the public sector on both scales.

There was a significant difference regarding medical specialty for the CARE measure (F (2,942) = 7,426 –p<0,005), but not for the JSPPPE (F (2,942) = 2,904 –P>0,05).

Subsequent consultations resulted in a higher score for empathy on the CARE measure, but no difference was observed with the JSPPPE scale when compared to initial consultations.

### Associations between the patients’ and the physician self-assessed empathy measures

As the number of patients per doctor differed we averaged the patients’ responses to their particular physician before conducting the Pearson correlation analysis. All correlations between patients’ and physicians’ perspectives were not significant, with one exception (Table 5). The score of the JSPPPE positively and significantly correlated with the sub-score of the Perspective Taking dimension of the JSE. Also, the outcomes regarding the intra-class analyses showed a low agreement between the empathy perception of the physician and the patient (ranging from 0,151 to 0,197). The exception was item 1 from JSPPPE with items 3, 6 and 9 from JSE in which the analyses did not have enough variance.

**Table 5:** Pearson correlations between empathy measurements: Physicians’ and patients’ perspectives.

Physicians’ Perceptions (n = 51)		Patients’ Perceptions’ (n = 945)	
		JSPPE	CARE
**JSE**	Perspective Taking	0,301*	0,04
	Compassionate care	0,07	0,03
	Standing in the Patient's Shoes	0,10	-0,07
	Jefferson Total	0,23	0,01
**IRI**	Perspective Taking	0,14	-0,09
	Empathic concern	0,06	0,14
	Personal Distress	-0,02	-0,04
	Fantasy	0,08	0,04
	IRI Total	0,12	0,02

* p < 0.05. Note: Perspective Taking and Empathic concern are other-oriented dimensions of IRI, while Personal Distress and Fantasy are self-oriented.

There were no significant correlations between the total scores and the sub-scores of IRI and JSE.

## Discussion and conclusion

### Discussion

The present study was developed with the underlying premise that patient involvement is vital to evaluate physician’s empathy. Our study indicates that the JSPPPE and CARE scales may capture different aspects of how patients perceive their physician’s empathy but that they share a common element. Both are validated measures which should be more widely employed.

Our study also revealed that contextual factors influenced patients’ perspectives of the empathy they received. We found a small but significant difference in CARE measures among different medical specialties, which was not found with the JSPPPE. CARE was also able to capture differences between empathy measurements from initial and subsequent consultations. It suggests that the CARE measure can capture more subtle nuances of patients’ interactions with their doctors, confirming its value to address relational components of empathy. Another interesting aspect of our results is the influence the sector (public vs private) had on patients’ scores. In Brazil, the Health System is divided into a public sector, supported by the government, and a private sector, maintained by private profit-driven Health Insurance Companies. Our results show that patients in the private sector tend to give higher empathy scores to their doctors. Two interpretations are possible: the doctors are consciously or unconsciously modulating their behavior, or there are other elements influencing patients scores beyond the direct interaction with their doctors. Regarding the first possible interpretation, a study with German doctors showed a positive impact of financial incentives on patients’ perceptions of physicians’ empathy measured by the CARE scale, which is aligned with our findings [26]. Considering the second possibility, a similar survey in Argentina showed opposite results, with patients scoring higher physicians from the public sector. The authors of this second study hypothesized that patients were expecting less from the doctors working in the public sector [28]. It could suggest that different cultural expectations regarding the public or private health systems can interfere in patient’s perceptions.

This study involved patients and doctors exclusively from one institution and nationality. Extrapolations of findings to other cultures need to be cautious, as patient definitions and classifications of empathy are culturally sensitive. Indeed, patients from different cultures can have disparate expectations regarding an empathetic doctor and doctors themselves can vary their understandings about how to demonstrate empathic concern or shared understanding [41]. In addition, the interpretation of the instrument items may have cultural nuances that the study did not expose, as that went beyond the predetermined goals. For instance, the item “How was the doctor showing care or compassion” (CARE scale) can be differently understood by a European, South American or Asian patient. Subsequent research should clarify the influence of cultural specificities in our findings.

An important objective of our study was to evaluate associations between widely used physician self-assessed empathy measures (JSE and IRI) and patient measures (JSPPPE and CARE) collected from the respective patients. The main finding was that there were no correlations between physician and patient measures. Overall, this study confirmed that self-assessed physician measures: JSE and the IRI, did not accord with the perceptions of their patients about their empathy as collected with the CARE and the JSPPPE. The main implication of this study is the confirmation that inferences about physician empathy based on self-assessment measures should not be taken as representative of patient perceptions.

The correlation of JSPPPE with the Perspective Taking dimension of JSE is in accordance with its development, which aimed the cognitive aspect of empathy [33]. Both instruments were developed by Hojat et al and use similar wording [29]. On the other hand, the CARE items were developed based on in-depth interviews with patients and further refined through interviews with patients, physicians and experts [34,35]. The CARE items aim to capture the internal atmosphere of the consultation; valuing empathy in the context of listening, reassuring and planning, from a patient perspective [34]. The lack of correlation of CARE and self-assessed measures of empathy gives insight into how inefficient self-assessment could be in capturing patients’ subjective experiences.

### Conclusion

Patients’ assessment of physicians’ empathy must be valued in empathy research and also in clinical care evaluation. More research is needed to investigate better how the different components of empathy and the various elements of the clinical encounter influence patient care and satisfaction. Physicians’ self-assessment of their own empathy is insufficient to evaluate the complexity of the interaction between doctors and patients. Insights from the empowerment of patients can give opportunities to develop training strategies to physicians willing to improve their clinical interactions with patients. Health care providers can also use this data to bring the multi-professional team together to discuss how to implement changes in patient care that could foster a patient-centered approach. Future research should explore what patients demand for their physicians’ behaviors and attitudes to acknowledge them as empathic doctors.

### Practice implications

Our data corroborate the understanding that empathy is a complex construct that should be evaluated through different lenses, depending on the goal of the evaluation process. If the intention is to create awareness among clinical doctors or medical students about the clinical value of empathy, self-assessment may represent a good strategy, providing there is awareness of the advantages and disadvantages of the instruments used. If, however, the purpose is to improve clinical care, then education and other interventions based on self-assessment instruments may not be enough because self-assessment cannot capture the reality as perceived by patients. As such, the curricular design for teaching and learning empathy and the international studies about medical students’ empathy development should be revisited, and there are implications for curricula and for teaching and learning empathy, of international studies about the development of undergraduate medical students’ empathy [42] and of studies evaluating the impact of programs to enhance empathy [43] should be revisited. Not only is selecting and applying the appropriate instrument to the research or practical question under investigation important but also, where ever possible, including patients is crucial.

Our contribution to the field is to show that the patient scales JSPPPE and CARE could be measuring different components of empathy and that other elements of the clinical encounter affects patients’ perceptions of their physicians’ empathy.

### Limitations

Unfortunately, our study was not designed to identify and to discriminate all the possible elements that could interfere with patients’ perceptions. However, we could hypothesize that time spent on consultation, time waiting for consultation, the general comfort of the environment, interactions with other health care professionals, the sense of the dignity of the process of care, and also heuristic bias related to the act of paying for the consultations are all possible factors. Further research is necessary to clarify these relations and how they can affect patients’ perceptions of physicians’ empathy, so allowing a more meaningful interpretation of data originated from these measurements. Also, our data were collected in a single center which can prevent generalization of the results. Although the number of interviewed patients was adequate, the number of doctors participating was small.

## Supporting information

S1 Data (XLSX)
